# Improving the Quality of Human Upper Urinary Tract Specimens by Cryobiopsy

**DOI:** 10.3389/fonc.2022.810367

**Published:** 2022-02-11

**Authors:** Jan T. Klein, Axel John, Lars Bohnert, Markus D. Enderle, Walter Linzenbold, Christian Bolenz

**Affiliations:** ^1^ Department of Urology, University of Ulm, Ulm, Germany; ^2^ Department of Research and Basic Technologies, Erbe Elektromedizin GmbH, Tuebingen, Germany

**Keywords:** upper tract urinary cancer, cryobiopsy, biopsy devices, UTUC, new biopsy devices

## Abstract

**Objective:**

The quality of histopathological specimens obtained from the upper urinary tract with conventional flexible ureterorenoscopic biopsy needs to be improved. We investigated the feasibility and biopsy quality of specimens obtained by cryobiopsy, compared with standard ureterorenoscopic biopsy techniques in a human ex vivo model.

**Materials and Methods:**

Human ureters obtained from nephrectomy specimens (N=12) were dissected and canulated with an ureteral access sheath. Ureterorenoscopic biopsies were randomly obtained from different sites of the renal pelvic caliceal system using different types of instruments. The performance of two newly developed flexible cryoprobes with outer diameters of 1.1 mm (CB11) and 0.9 mm (CB09) was compared with that of the biopsy forceps(FB) and Bigopsy®(BiG) and two different Dormia baskets N‐Gage (NG) and Zero‐Tip (ZT). We assessed the feasibility of the various biopsy techniques based on the number of biopsy attempts needed to obtain macroscopically discernible biopsies. The specimens were examined histopathologically for size, biopsy quality, presence of various artifact types, and representativeness.

**Results:**

Biopsies taken with the cryoprobes showed a higher biopsy quality than biopsies taken with the comparative instruments. The CB11 provided significantly larger biopsies than forceps biopsies and also than biopsies with ZT. The CB09 was able to collect larger samples when compared with the FB and BiG biopsy forceps. There were no significant differences in artifact area, except for the CB11 cryoprobe compared with the NG. To clarify the results a subdivision of larger or smaller than 20% artifact area was performed. A significant difference was found between CB11 and the forceps biopsies, as well as between CB11 and NG and ZT in favor of the cryoprobe. The representation of the histopathological sample was also determined. Biopsies taken with CB11 were more representative compared with forceps biopsies BiG and FB and basket biopsies NG and ZT.

**Conclusions:**

In a standardized comparative ex vivo setting, larger biopsies were obtained by using the cryobiopsy technique with the CB11 probe. Qualitatively, cryobiopsy specimens were overlaid by fewer artifacts and a higher biopsy quality was achieved in histopathologic examination compared with standard instrumentation. Further stepwise development will transfer the promising cryobiopsy technique into the clinical setting.

## Introduction

Upper urinary tract carcinoma (UTUC) is a relatively rare entity, representing 5-10% of all urothelial carcinomas (1). UTUC has similar morphology as bladder carcinomas and almost all UTUCs are urothelial in origin. Surprisingly, UTUCs are much more frequently invasive compared to urothelial carcinoma of the urinary bladder at the time of diagnosis (1). Organ-preserving treatment strategies have been developed for many other tumor entities and are also available for UTUC under certain conditions (1). The decision for or against a kidney-preserving therapy procedure is based on a correct histological classification of the tumor into low- or high-risk tumor. Histopathological grading and staging are essential for determining treatment options and prognoses. Without biopsies, the diagnosis rate is only 50-60%. With additional ureterorenoscopic biopsies, the rate is 80-90%. The biopsy procedure used must allow for a differentiation between these two groups. The type of biopsy technique influences the diagnosis. The combination of forceps biopsy and Dormia basket shows the best biopsy quality to date (1). However, despite current biopsy instruments and procedures, a considerable number of biopsies are proven to be too small and of insufficient diagnostic value due to artifact overlay. This means that sampling is prone to errors and does not allow a reliable diagnosis in every case. In a quarter of all cases, adequate grading cannot be performed because the tissue sample is too small (1-2mm) or crush artifacts and associated disturbances of the tissue architecture occur (2). Restaging or regrading after radical nephroureterectomy is required in approximately one third of all cases (3). It was reported that biopsies <1mm may not allow for reliable diagnosis (4). The quality of the preparation depends on the type of biopsy forceps used. Novel frontloading biopsy forceps are superior to the classic backloading ones, especially for flat or sessile lesions (5). To overcome these limitations, the arsenal of biopsy instruments is expanded to include the “Dormia baskets”. For papillary lesions, basket biopsy is particularly suitable (5).

There is a trend toward understaging; for example, 45% of tumors initially classified as Ta have to be graded to pT1 or higher postoperatively. Some tumor entities, such as carcinoma *in situ*, almost completely escape diagnoses. Thus, histopathologic diagnosis needs much improvement (6).

Improving the diagnostic accuracy is currently subject to several research projects. For this purpose, non-invasive diagnostic techniques such as narrowband imaging, optical coherence tomography, confocal laser microscopy or photodynamic diagnostics have been investigated. These technologies improve the optical detection of tumor tissue and allow an in-situ assessment of tumor tissue. However, this does not affect the biopsy quality itself.

The cryoprobe itself is already used in other medical fields, such as in the ablation of tumors or in the treatment of atrial fibrillation (7, 8). Cryotechnology has been successfully and diagnostically used on humans in pulmonology for transbronchial lung biopsies in suspected interstitial lung diseases (9). Larger, higher quality samples with fewer complications can be obtained from human tissue (10–12). The sample size depends on the type of tissue, the probe diameter, the application time and the contact pressure (13). In addition, despite the freezing process, molecular markers can be retained in the tissue, which indicates that the tissue integrity remains intact (14). Ureterorenoscopic cryobiopsy could decisively improve the quality of the histopathological biopsate obtained, thus making a significant contribution to organ preservation in UTUC.

We have previously shown that cryobiopsy is feasible in the upper urinary tract of porcine kidneys. In this animal study, larger samples with a low artifact load have been successfully obtained (15). In addition, compared to the other biopsy devices, the cryoprobe did not produce crush artifacts and consistently yielded pathologically assessable samples. There are no data in the literature on the use of cryobiopsy in the human upper urinary tract. In order to evaluate the feasibility and value of cryobiopsy in this field, the use of the cryoprobe was performed in an ex vivo experiment on the human urinary tract.

## Materials and Methods

### Study Design and Patient Selection

The study had a controlled, prospective, single-blinded, monocentric design. Patients for whom removal of the kidney was indicated for various reasons were enrolled in the study. Patients with renal tumors and patients with high-risk UTUC requiring nephrectomy were included. To determine the feasibility of cryobiopsy, the sample volume, and the artifact area, we also included patients with chronic kidney disease and clinically non-functioning kidneys (e.g., chronic pyelonephritis or shrunken kidney) that needed to be removed for appropriate indications, such as hypertension, chronic urinary tract infection, or recurrent pain. In total, N=12 patients were enrolled in the study.

Patients with florid local renal inflammation, systemic inflammation in the context of sepsis or acute renal trauma who required emergency nephrectomies and transplant kidneys were excluded because the pathomorphological changes expected in these patients could have confounded the results. In addition, patients under 18 years of age, pregnant patients and patients unable to give their own consent were excluded. This study was approved by the local ethic review board.

### Cryoprobe

Two disposable types of cryoprobes (Erbe Elektromedizin GmbH, Germany) were used in this study: one with an outer diameter of 1.1 mm (CB11), which is already available on the market, and one prototype of a new cryoprobe with an outer diameter of 0.9 mm (CB09). The cryoprobes are connected to a standard carbon dioxide gas pressure cylinder *via* the control unit, the ERBEKRYO2 device (Erbe Elektromedizin GmbH, Germany), which are inserted in retrograde into the ureterorenoscope. Activation and activity duration, and thus ice ball formation at the cryoprobe tip, can be determined *via* a foot pedal connected to the control unit.

The principle of the cryoprobe is based on the Joule-Thomson effect, which describes a temperature reduction through the sudden decompression of a gas (here CO_2_). The probe itself consists of an outer tube, an inner lumen and an outer lumen. In the inner lumen, the compressed gas flows at a pressure of approximately 55 bar to the tip of the probe, where an abrupt decompression of the gas occurs resulting in the cooling of the metal probe tip. The gas flows through the outer lumen back to the control unit and is released to the room.

The metal tip is brought into contact with the tissue to be biopsied. The activated metal tip of the cryoprobe cools the tissue. Very fine ice crystals form initially, and on further activation, an ice ball encloses the tissue undergoing biopsy. The ice crystals cause the tissue to adhere to the metal surface of the probe, so that the sample adheres to the metal tip by adhesion and can be released from the tissue dressing with a jerk of the probe.

Two factors determine the sample size: first, the probe contact pressure, which can be determined manually by the surgeon. Second, which is the most influencing factor that can be set on the control unit, is the freezing duration.

To obtain optimal biopsy sizes, it is important that the ice ball surrounding the tissue is not too large, so that the ice ball’s adhesion to the tissue is weaker than to the probe; otherwise, this would cause the ice ball to detach from the probe tip. In addition, the sample must be large enough to allow for histopathological evaluation. We have determined these parameters to be optimal at a sampling time of 7 seconds for both cryoprobes. In this case, cell morphology is preserved despite the freezing process and the sampling can reliably succeed.

The timer of the ERBEKRYO device starts as soon as the pedal is pressed and can be read on a connected display or can be detected acoustically. With the cryoprobe, biopsies can be taken both tangentially or frontally. After the specified activation time, the probe and endoscope are pulled out as a unit with a jerk movement through the ureteral access sheath, as the sample adhering to the probe is too large to fit through the working channel of the endoscope. After the extraction process is complete, the cryoprobe is deactivated and the ice ball forms back. The biopsy detaches from the metal tip and can be preserved in a formalin-containing container for pathology.

### *Ex Vivo* Model Human Nephrectomy Specimen

**Figure 1:**
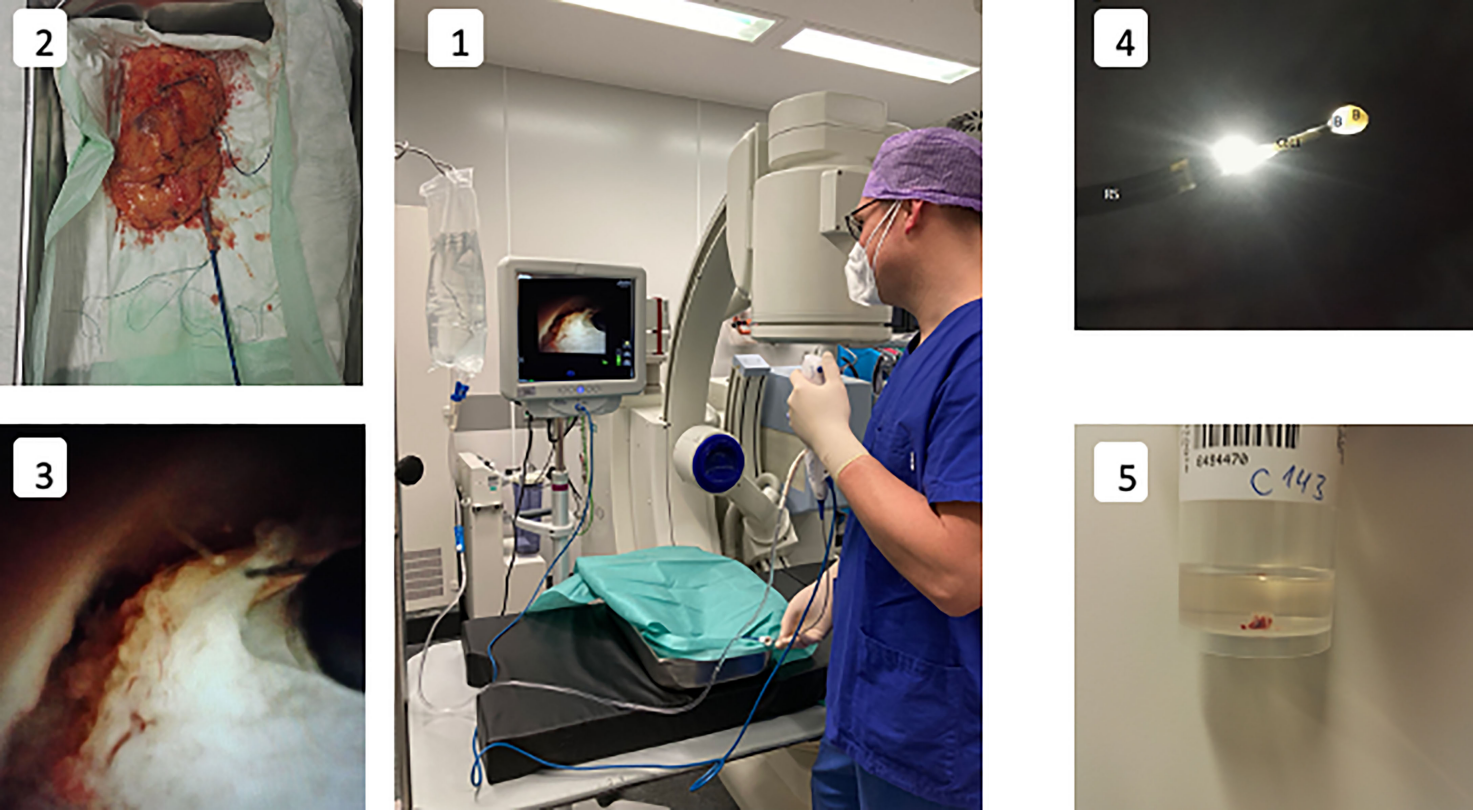
Experimental setup and procedure. 1: Experiment setup-surgeon performing a endoscopic biopsy under irrigation with sodium chloride solution utilising a digital ureterorenoscope. 2: Kidney specimen with attached ureter cannulated with an access sheath. 3: Endoscopic visualization of cryoprobe (CB11) in the renal pelvicocaliceal system almost in contact with the tissue, shortly before activation. 4: Ureteroscope (RS) after biopsy with the cryoprobe (CB11) with ice ball (IB) adhering to the tip of the cryoprobe and a biopsy specimen (B) enclosed within. 5: Container for the biopsy specimen. The formalin solution cotains a macroscopically visible cryobiopsy specimen.

**Figure 2:**
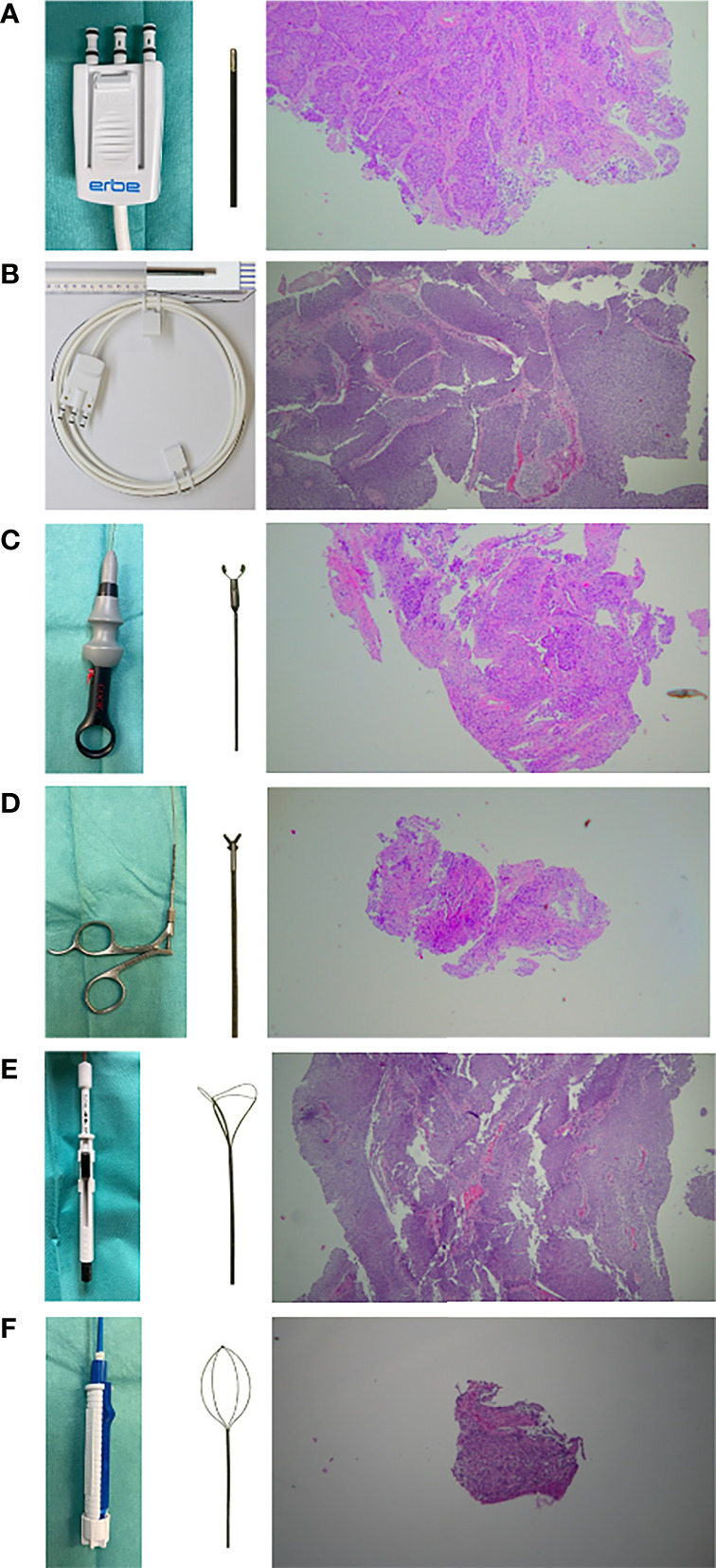
Overview of the instruments used and exemplary representation of the histological specimen (HE strain, 20x magnification) obtained. **(A, B)** cryoprobe with outer diameters of 1.1 mm and 0.9 mm (Erbe Elektromedizin, Tuebingen, Germany). (CB11) & (CB09) **(C)** frontloading disposable biopsy forceps for the upper urinary tract (BIGopsy  No. BLB-024115, Cook Medical, Bloomington, IN, US). (BiG) **(D)** reusable biopsy forceps for flexible ureteroscopy (No. 829.601, Richard Wolf GmbH, Knittlingen, Germany). (FB) **(E)** tipless front grasping Dormia basket N-Gage (No. NGE-022115, 2.2F Cook Medial, Bloomington, IN, US). (NG) **(F)** tipless side grasping Dormia basket: Zero-Tip (No. 390105; UPN M0063901050, Boston Scientific, Marlborough, Massachusetts, US). (ZT).

The nephrectomy specimen was harvested. One specimen was a nonfunctional hydronephrotic kidney, seven kidneys had been removed for locally advanced renal cell carcinoma, and four kidneys had been removed for upper tract urothelial carcinoma. The ureter was dissected, dilated and a ureteral access sheath (Navigator®, 13/15 Ch, 28cm, (Boston Scientific, Marlborough, Massachusetts, US) was inserted and fixed. In order to create an optimal basis for comparison, we always used a brand-new disposable flexible ureterorenoscope LithoVue® (Boston Scientific, Marlborough, Massachusetts, US). The biopsies were performed under 0,9% saline irrigation with the different devices in a randomized order. The experimental setup is shown in **Figure 1**. The following biopsy instruments were used: two different disposable cryoprobes with outer diameters of 1.1 mm (CB11) and 0.9 mm (CB09) (Erbe Elektromedizin, Tuebingen, Germany), reusable biopsy forceps (FB) for flexible ureteroscopy (No. 829.601, Richard Wolf GmbH, Knittlingen, Germany), and a frontloading disposable biopsy forceps for the upper urinary tract (BIGopsy^®^ No. BLB-024115, Cook Medical, Bloomington, IN, US). Additionally, two different tipless Dormia baskets were used: a front grasping N-Gage® basket (No. NGE-022115, 2.2F Cook Medial, Bloomington, IN, US) (NG) and a side grasping Dormia basket: Zero-Tip® (No. 390105; UPN M0063901050, Boston Scientific, Marlborough, Massachusetts, US) (ZT) (**Figure 2**). This corresponds with the standard instruments for biopsies in the upper urinary tract. For data processing, a study ID was determined for each patient and recorded in an Excel spreadsheet in CRF format, which ensured the pseudonymization of the patients. Only when the data were complete and representative were they entered into the study database. A histopathological examination was performed using a standard microscope (Axio Vision LE REL 4.4; Carl Zeiss Microimaging, Göttingen, Germany). Up to three biopsies were obtained with each probe. The number of biopsy attempts and potential biopsies obtained per instrument, per kidney in all twelve kidney specimens examined are shown in **Table 1**.

**Table 1:** Possible numbers of biopsy attempts and possible biopsies to be obtained per instrument, per kidney, for all twelve kidney specimens examined including the trial results.

Parameter	Minimum	Maximum
***Possible number of attempts***		
per device per kidney	3	9
using all six devices per kidney	3*6 = 18	9*6 = 54
in N=12 kidneys	3*6*12 = 216	9*6*12 = 658
***Possible number of biopsies***		
per device per kidney	0	3
using all six devices per kidney	0	3*6 = 18
in N=12 kidneys	0	3*6*12 = 216
***Trial results (N=12 kidneys, 6 devices)***		
Number of attempts performed	266	
Number of biopsies obtained	175	

We assessed whether it was generally possible to obtain a biopsy with the different devices, regardless of the quality or quantity of the biopsy. “Yes” meant that a macroscopically identifiable sample could be obtained within 3 attempts and hence feasible, “no” meant that a sample could not be obtained. This was assessed immediately after each biopsy.

The biopsy reliability was determined by the number of times a biopsy had to be performed before a sample was successfully obtained. The number was recorded. The tissue sample was considered “not obtained” if macroscopic tissue could not be obtained, even on the third attempt. Subsequently, whether a sample could already be successfully obtained was analyzed during the first biopsy attempt.

### Histopathologic Evaluation

All obtained biopsy specimens were fixed in 4.5% neutral buffered formalin, processed into paraffin blocks, and stained with hematoxylin and eosin. Subsequently, the samples were analyzed by the reference pathologist (TB) using a predefined histology score for the following parameters: Total area of the specimen in mm^2^, biopsy quality score, percentage of artifact area and presence of squeeze artifacts. The biopsy area was determined based on the length and width of the sample. There is already a scheme for assessing quality from pneumology (13, 16), which has been adapted in more detail for urology (15). The histopathologic quality score is evaluated using an ordinal scale and was also assessed by an experienced pathologist (TB). The artifact area was defined by the area fraction of the artifacts in relation to the cross-sectional area of the sample. A specimen was considered representative if, first, it was large enough to perform a histopathologic examination, second, it had an artifact score of 0-1, and third, if the quality was rated at least at 2.

### Statistical Analyses

Statistical analysis of the samples was performed using IBM SPSS Statistics version 27 for Windows (released 2020, Armonk, NY, IBM Corp.). The chi-square test was used to test whether two categorically distributed variables were statistically different. We used this test to determine whether the biopsy was successful in the first attempt and to examine whether it was possible to obtain samples at all. We also used it to calculate whether the specimens differed significantly in their artifact area, by dividing them into specimens with an artifact area greater than 20% and also less than 20%. The chi-square test was used to test representativeness for statistical significance. A one-way analysis of variance (ANOVA) with Welch correction was used for unpaired data from the Gaussian distribution with unequal standard deviation, with a Dunnett multiple comparison correction for multiple comparisons as *post hoc* analysis. This was used to compare the cross-sectional area of the samples. The Kruskal-Wallis H test was used to test rank-based nonparametric values between two or more groups of independent variables. In this case, our post-hoc nonparametric test was the Dunn multiple comparison test. We used these tests to examine the percent artifact area and biopsy score. Descriptive statistics were reported using standard deviation and mean, or median and range for nonparametric distribution. Rank correlation was calculated using the Spearman rank correlation coefficient. Plots were presented using the mean ± the standard error of the mean.

## Results

No difference was found between CB11 and CB09 when directly compared in any of the factors examined. The analysis focused on the performance of the cryoprobes in comparison to the other devices. **Table 2** shows the summary of the results for the different factors.

**Table 2:** Comparison of the parameters: feasibility, reliability, mean biopsy area, mean artifact area, artifact scores, pathology score and representativeness score of the different devices.

	Feasibility in 3 attempts in %	Reliability biopsy on first attempt in %	Mean biopsy areaIn mm^2^(SD)	Mean artifact area in %	Artifact score 0-1 in %	Artifact score 2-3 in %	Patho score mean AU	Rep score In %
CB11	83	63.9	14.5 (±9.7)	11	89.7	10.3	4.9	89.7
CB09	80.6	63.9	12.7 (±6.0)	19	77.8	22.2	4.7	77.8
BiG	100	66.7	4.3 (±5.8)	24	69.3	30.7	3.2	80.6
FB	88	50	1.4 (±2.1)	28	65.2	34.8	3.6	60.9
NG	63	33.3	9.5 (±11.6)	48	53.3	46.7	3.3	53.3
ZT	72	36.1	6.7 (±7.9)	33	59.1	40.9	2.9	54.5

All devices were listed on the y-axis: cryoprobe 1.1mm, cryoprobe 0.9mm, disposable biopsy forceps BIGopsy^®^, standard biopsy forceps, Dormia basket Cook-N-Gage and Dormia basket Zero-Tip. SD, standard deviation.

**Figure 3:**
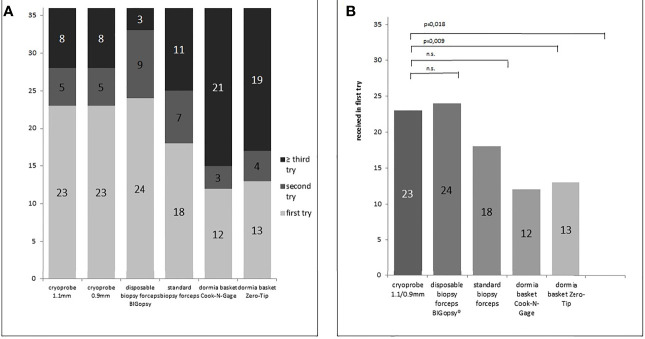
Number of biopsy attempts per biopsy device **(A)** and number of sample already received in the first biopsy attempt **(B)**. P values in B determined via chi-square test (n.s. = not significant). Samples were collected using the different biopsy devices (disposable biopsy forceps BIGopsy®, standard biopsy forceps, Dormia basket Cook-N-Gage and Dormia basket Zero-Tip-x-axis).

Biopsies were obtained with each instrument. The cutoff was set at 3 biopsy attempts. A total of 175 histologically evaluable biopsies were obtained from 266 biopsy attempts (**Table 2**). Forceps biopsies were superior to the other techniques, with the BiG being superior to all other instruments tested in terms of biopsy collection feasibility. 36/36 biopsies (Bx) were taken with the BiG (100%) and 31/36Bx with the FB (88%). Both cryoprobes were slightly less effective. 29/36 Bx (80.6%) were retrieved with the CB09 and 30/36 Bx (83%) with the CB11. The least efficient technique was the Dormia basket biopsy. 23/36 (63%) of biopsies were obtained with the NG Dormia basket and 26/36 (72%) with the ZT (**Figure 3**).

**Figure 4:**
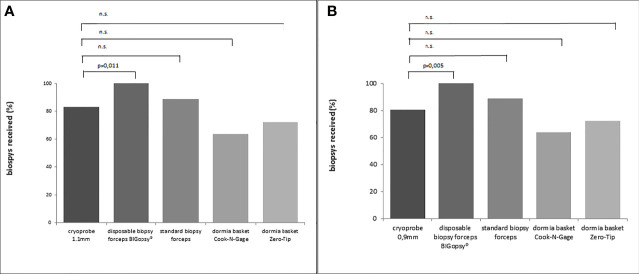
Percentage x/n from actual biopsies obtained (x) vs. number of attempts (n). In **(A)** cryoprobe 1.1mm vs other devices (disposable biopsy forceps BIGopsy®, standard biopsy forceps, Dormia basket Cook-N-Gage and Dormia basket Zero-Tip-x-axis). In **(B)** cryoprobe 0.9mm vs other devices. P values were determined by chi-square test (n.s., not significant).

At 66.7%, the BiG was the most efficient device for obtaining a biopsy in the first bite, followed immediately by the two cryoprobes. Both CB09 and CB11 proved to be almost as efficient as the BiG, with 63.9% biopsies in the first attempt. The FB took the first bite in half of the cases (50%), while the Dormia baskets NG (33.3%) and ZT (36.1%) proved to be less efficient than forceps biopsies. (**Figure 4**)

**Figure 5:**
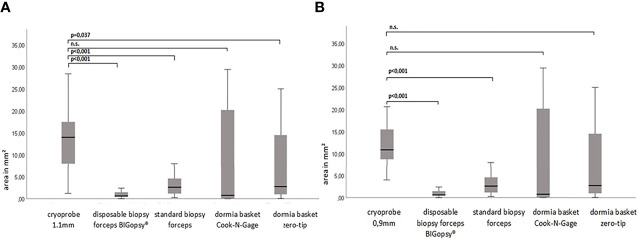
The biopsy size of the paraffine in mm^2^. P-values via Welch ANOVA for variance inhomogeneity with Dunn's multiple comparison of means. ANOVA, analysis of variance, Graph: median ± 95% confidence interval (n.s. = not significant). In **(A)** cryoprobe 1.1 mm vs. other deviced (disposable biopsy forceps BIGopsy®, standard biopsy forceps, Dormia basket Cook-N-Gage and Dormia basket Zero-Tip- x-axis. In **(B)** cryoprobe 0.9mm vs other devices.

The average area of the biopsies obtained with the cryoprobes CB11 and CB09 yielded the largest samples with 14.5 mm^2^ and 12.7 mm^2^, respectively. The basket biopsies were also of sufficient size, at NG 9.5 mm^2^ and 6.7 mm^2^. Relatively little tissue could be obtained with forceps biopsies: BiG 4.3 mm^2^ and FB 1.4 mm^2^. The CB11 yielded significantly larger biopsies than the FB (p<0.005), the BiG (p<0.005) and the Dormia basket ZT (p=0.037). The CB09 collected significantly larger samples compared to the FB (p<0.005) and the BiG (p<0.005) (**Figure 5**).

**Figure 6:**
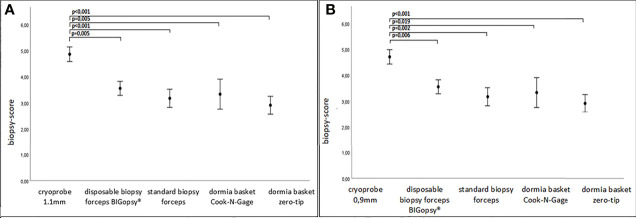
Biopsy score in A.U (arbitrary unit). P values were collected via rank based nonparametric Kruskall-Wallis test, post hoc test via Dunn’s multiple comparison of means. Graph: mean ± standard error of the mean (1x). In **(A)** cryoprobe 1.1mm vs other devices (disposable biopsy forceps BIGopsy®, standard biopsy forceps, Dormia basket Cook-N-Gage and Dormia basket Zero-Tip-x-axis). In **(B)** cryoprobe 0.9mm vs other devices.

Consequently, the quality of CB11 and CB09 were superior to all other devices in terms of biopsy quality (**Figure 6**).

**Figure 7:**
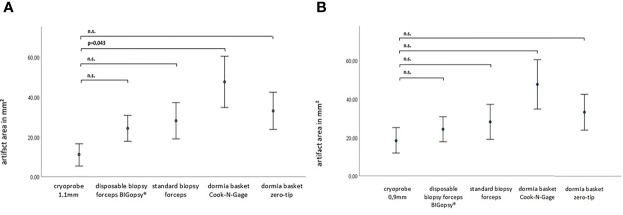
The artifact area within the paraffine section in mm^2^. P-values via Welch ANOVA for variance inhomogeneity with Dunn's multiple comparison of means.ANOVA, analysis of variance, Graph: means ± standard error of the mean (1x) (n.s. = not significant). In **(A)** cryoprobe 1.1 mm vs. other deviced (disposable biopsy forceps BIGopsy®, standard biopsy forceps, Dormia basket Cook-N-Gage and Dormia basket Zero-Tip- x-axis. In **(B)** cryoprobe 0.9mm vs other devices.

The average area of the biopsy specimen overlaid with artifacts was determined. Interestingly, the pronounced artifact overlays were seen in the biopsies obtained with the Dormia baskets ZT (33%) and NG basket (48%). This was followed by the forceps biopsies FB (28%) and BiG (24%) and the least artifact overlays were obtained from the cryo-tissue sample CB11 (11%) and CB09 (19%). Due to a wide distribution of measured values, there were no significant differences between the cryoprobes and the other devices, with the exception of the CB11 compared to the Dormia basket NG (**Figure 7**).

**Figure 8:**
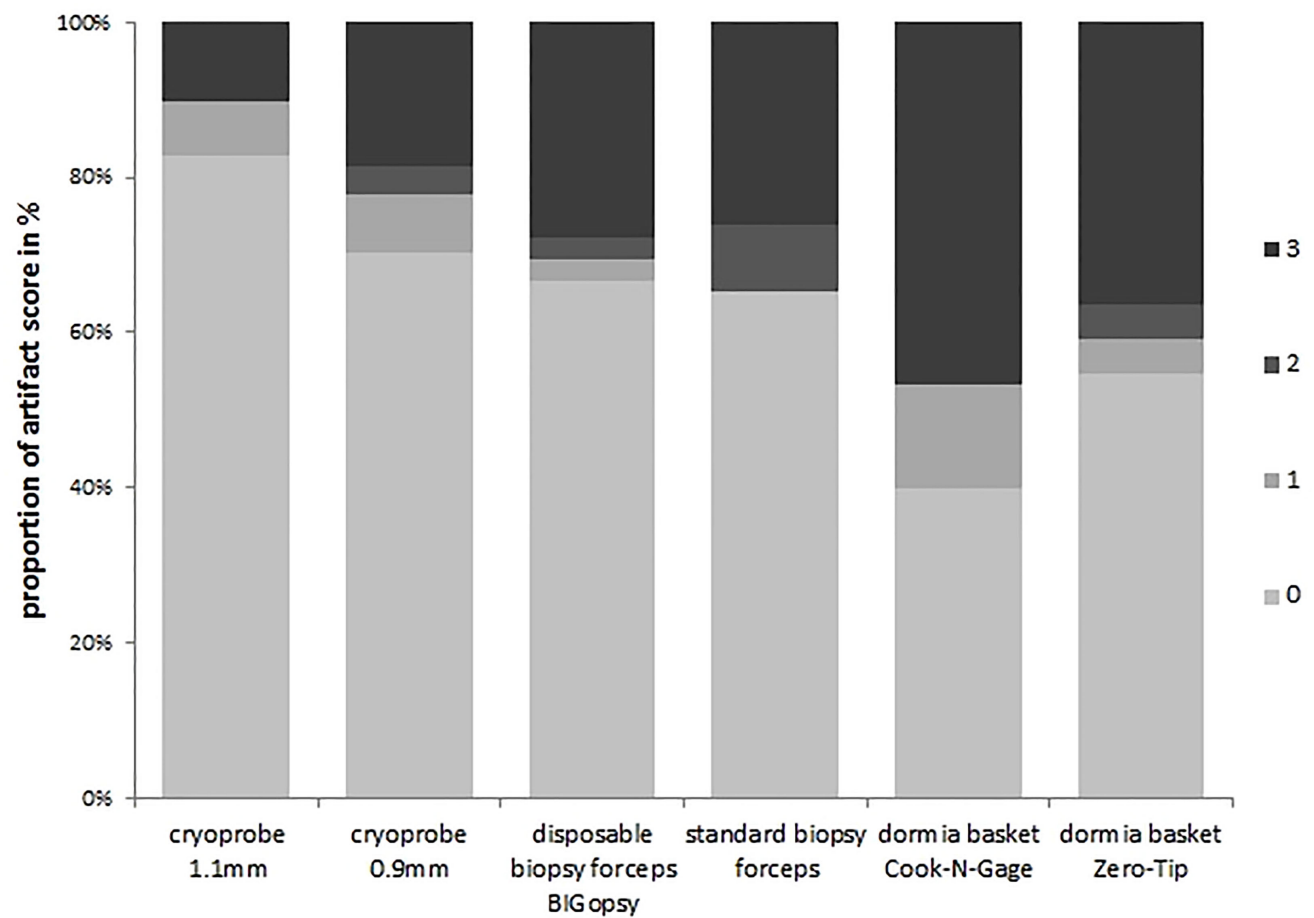
Percent artifact scores per category were determined by dividing the number of biopsies per artifact score category by the number of total biopsies. All biopsies were previously assigned to an artifact score category.The devices were listed on the x-axis:cryoprobe 1.1mm, cryoprobe 0.9mm,disposable biopsy forceps BIGopsy®, standard biopsy forceps, Oormia basket Cook N-Gage and Oonnia basket Zero-Tip.

**Figure 9:**
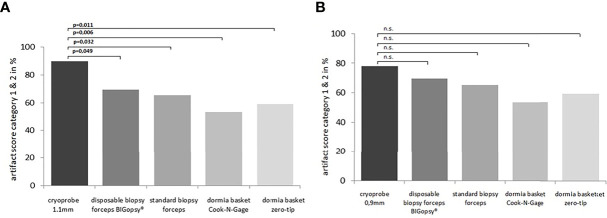
The relevant number of biopsies on the total biopsies falling into artifact score category 0 or 1 [score in A.U.(arbitrary unit)] were compared among each other. In **(A)** cryoprobe 1.1 mm vs.other devices (disposable biopsy forceps BIGopsy®, standard biopsy forceps, Dormia basket Cook N-Gage and Dormla basket Zero-Tip-x-axis). In **(B)** cryoprobe 0.9mm vs.other devices. P values were determined by chi-square test (n.s. = not significant).

A subgroup analysis was performed to identify the differences more clearly. In the subgroup analysis, the artifact area was categorized as less than (artifact score 0 & 1) and larger than 20% (artifact score 2 & 3). Tissue was the least altered by the cryoprobe removal, resulting in a low artifact score (0–1). CB11 (89.7%) and CB09 (77.8%) preserved tissue best with a low artifact score. Forceps biopsies with FB (65.2%) and BiG (69.3) showed inferior tissue preservation. Biopsy with the Dormia basket was the most overshadowed by higher grade artifacts, but was still able to achieve an artifact score of 0-1 in > 50% of cases: NG (53.3%) and ZT (59.1%). The statistical analyses showed that a significant difference was observed between the CB11 and the BiG (p=0.049), the FB (p=0.032), and the NG (p=0.006) and ZT Dormia baskets (p=0.011) in favor of the CB11. This trend was also observed for CB09, but the values were not statistically better (**Figures 8** and **9**).

**Figure 10:**
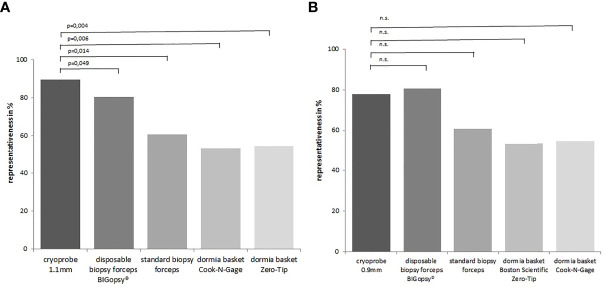
The proportion of representative samples in the total biopsy number. Representative = sufficient size + artifact score category 0/1+ biopsy score 2 6. In **(A)** cryoprobe1.1 mm vs. other devices (disposable biopsy forceps BIGopsy®, standard biopsy forceps, Dormia basket Cook-N-Gage and Dormia basket Zero-Tip-x-axis). In **(B)** cryoprobe 0.9 mm vs other devices. P value-s were determined by chi-square test (n.s. = not significant).

Taken together, the CB11 achieved the best results compared to the other instruments with representative tissue samples in 90% of cases. The superior quality of samples obtained with the CB11 proved statistically significant compared to the BiG (p=0.049), FB (p=0.014), NG (p=0.006) and ZT (p=0.004). The CB09 with 78% representative samples did not prove to be more representative than all the other devices used (**Figure 10**).

## Discussion

Preliminary studies in the ex vivo porcine model using CB11 showed that the ureterorenoscopic cryobiopsy of the upper urinary tract is feasible (15). In our ex vivo study of human tissue, cryobiopsy also proved feasibility and reliability comparable to forceps biopsy (BiG, FB), and was more reliable as a Dormia basket biopsy (NG, ZT). The flat lesions and scarless tumor-free tissues were also biopsied in the study, which explains the comparatively poor performance of the baskets, which are normally very effective for exophytic lesions.

A distinct advantage of cryoprobe biopsy is that flat lesions can be biopsied due to the possibility to contact the tissue tangentially and the size of the biopsy can be controlled by the activation time of the probe. In addition, the lack of forceps blades and the cold exposure of the tissue results in fewer squeezing artifacts and better preservation of the different tissue layers (15). For a successful biopsy an optimal tissue sample under ideal freedom of movement and perfect visibility is desirable.

The handling of cryoprobe-guided biopsy is relatively simple for the experienced endourologist. The probe is inserted in retrograde through the instrument channel. The tip of the probe is blunt enough not to damage the inside of the endoscope. The tip of the probe is clearly visible in the image and, in contrast to BiG, only obscures a fraction of the endoscopic monitor image compared to the FB, even more with the 0.9 mm cryoprobe prototype. Although there was nearly no difference between the two cryoprobes, the 0.9 mm probe was slightly more bendable and showed a better irrigation flow rate compared to the 1.1 mm (data not shown), which can be explained by the small outer diameter (15).

One disadvantage is, that the probe must be passed through an access sheath; there is debate as to whether this is a potential risk to the patient from a tumor biology perspective. However, the cryoprobe must always be taken together with the endoscope. Particularly in the case of biopsy in the lower calyx group with a steep calyx neck angle, a tissue enclosed ice ball may be sheared off the tip of the cryoprobe by the edge of the access sheath when the instrument is withdrawn. Theoretically, in these cases a secondary removal of the specimen using a dormia basket should be technically feasible.

The therapeutic spectrum of UTUC is diverse and associated with different risks and long-term consequences for the patient, depending on the method chosen. A purely visual ureteroscopic decision would misclassify 30% of tumors (17). Biopsy can increase the diagnostic rate to as high as 90% (18), but in biopsies taken with conventional biopsy techniques, only one in four specimens collected is diagnostic (19). If diagnosis is not possible due to incorrect biopsies, reinterventions are likely to occur with the renewed risk for patients of bleeding, ureteral perforation, tumor spillage or urinary tract infections. If the primary diagnosis is incorrect, necessary radical surgical therapy is delayed with a potentially worse outcome for the patient.

For low-risk carcinomas, kidney-preserving endoscopic interventions are the method of choice (20). However, correct staging is crucial (21). Rojas et al. demonstrated that 57% of biopsies were classified as understaged, which may lead to erroneous renal preservation procedures despite invasive tumors (22). A high proportion of biopsies still cannot be utilized. Al-Qahtani et al. listed 10 of 40 biopsies as too small and not evaluable. Tavor et al. also showed 25% non-evaluable biopsies, and in a study by Breda et al., 66 of 302 (21.8%) biopsies were not evaluable (6, 18, 19). Cryobiopsy with a high level of representativeness could be an alternative to reduce the error rate.

A key advantage of the cryoprobe is the ability for the surgeon to adjust the sample size by the activation time, and the contact pressure of the probe on the tissue (13, 23). This gives the surgeon, in contrast to all previous biopsy techniques, the possibility to control the biopsy size.

The question arises whether a larger sample volume actually affects histopathological assessment, and in this regard, the literature contains inconsistent results.

The concordance between the biopsy grading and the final histopathologic diagnosis on the explanted organ ranges from 67% to 93% (22, 24–27). Compared with 5.9/2.4F biopsy forceps, a Dormia basket, and standard 3.6/3F biopsy forceps, Lama et al. concluded that the size and quality of the specimen had no effect on grading (28). This was determined in a retrospective setting comparing specimens from 145 patients with subsequent nephrectomies. Rojas et al. also demonstrated that biopsy volume did not affect grading in 54 patients. Nevertheless, they showed that biopsy-based staging in particular has great potential for improvement (22). Consistently, Tavora et al. reported that in cases where a definitive diagnosis could not be made by experts, this was mainly due to biopsy size, cell architecture, and squeeze artifacts in 21.05% of the specimens (19). Williams et al. showed that larger tissue specimens are better able to demonstrate the architecture of the lesion, which seems particularly important in the diagnosis of low-grade tumors, which tend to have fewer memorable cytologic features than high-grade UTUCs (27).

Correct staging is often not possible, which, in turn, can complicate treatment decisions. Rojas et al., in a study of 51 patients, showed that correct staging was present in only 43% of patients (22). Brown et al. attempted to draw conclusions about staging from bioptic grading. They calculated positive predictive values for staging in the different grading groups. Patients with G3 UTUC had pT3 or higher tumors in 43% of the cases. Patients with high-grade biopsies had pT2 UTUCs in 66% of the cases. In contrast, 72% of patients with low-grade biopsies had a final histologic staging of less than pT2 (29). This was confirmed by another study with a PPV of 60% for high-grade biopsies (24). Brien et al. showed that of 74 patients with high-grade biopsies, 62% had a T2 tumor on the final histopathologic evaluation, and 68% with low-grade biopsies were actually classified as maximum T1 (30).

Overall, the trend is toward undergrading and understaging. In a direct comparison between biopsy and subsequent nephroureterectomy, tumors had to be upstaged in a study by Margolin et al. (31). For Guarnizo et al., in 10 of the 22 cases (45%) from pTa, pT1+ was elevated (range pT1 to pT3) (25). Smith et al. also had to upgrade 24 (43%) of their patients from low-grade, non-invasive to high-grade invasive (32).

Since staging is composed of tumor infiltration depth, among other factors, it is reasonable to assume that incorrect staging occurs because the surgeon tends to perform biopsies too shallowly, rather than too deeply, to avoid perforation and bleeding, particularly in the ureter. Stewart et al. suggest that the tunica muscularis in the upper urinary tract is more likely to be completely infiltrated because it has a very thin layer (33). However, a pTa tumor entails different therapy than a pT1+ tumor (34). Given these findings, it is clearly advantageous to use the instrument for the biopsy that provides the best quality and size of the tissue sample. Whether the cryoprobe also produces an improvement in staging needs to be investigated in further studies. For example, by using the cryoprobe in addition to the standard 3F biopsy forceps in the study by Guarnizo et al. and then comparing the results (25).

A recent meta-analysis by Nowak et al. evaluated the risk of developing a bladder tumor after ureterorenoscopic biopsy has been performed. An association was shown between biopsied and non-biopsy patients. The group of biopsied patients showed a higher incidence rate of tumors in the urinary bladder. Seeding of floating tumor cells may occur but the exact mechanisms for this have not been determined. The study collectives were too different for this. The application of various techniques, such as the use of a ureteral sheath as protection against recurrence in the urinary bladder, also showed no improvement (35). The extent to which cryobiopsy can circumvent this phenomenon is not yet clear. In contrast to other biopsy techniques, in cryobiopsy the biopsy specimen is tightly enclosed by the ice ball and cell spreading may thus be reduced. However, this still needs to be investigated in further studies.

Our study has some limitations. The number of samples and trials required was calculated in a power analysis before the start of the study and was determined with N=12 samples. However, a higher number of trials would increase the statistical power. The primary endpoint of the study was the feasibility of cryobiopsy of human urothelial tissue in the upper urinary tract and that endpoint was met. Not all nephrectomy specimens contained tumor tissue, so statistical power is limited. However, urothelial tumor tissue is more fragile compared to normal urothelium, so this effect can be considered marginal. Flat lesions and fibrous tissues were included in the study. This explains the reduced specimen size using the Dormia baskets. A questionnaire about the handling of the different probes would have been beneficial for evaluating the handling of the different instruments.

## Conclusions

Cryobiopsy on human urothelial tissue of the upper urinary tract is feasible. This includes urothelium, fibrous und tumor tissue. Significantly larger and higher quality samples compared to the standard armamentarium of upper tract biopsies could be obtained using both cryoprobes CB11. The cross-sectional area of the biopsy after the microscopy of the paraffin sections in mm² was significantly increased, compared with conventional biopsy methods. Furthermore, fewer squeezing artifacts were observed. Overall, our results indicated that cryoprobes, especially the CB11, can help improve diagnostics and therapy decision-making in UTUC. *In vivo* studies need to confirm the advantages of cryobiopsy in a clinical setting.

## Data Availability Statement

The original contributions presented in the study are included in the article/supplementary material. Further inquiries can be directed to the corresponding author.

## Ethics Statement

The studies involving human participants were reviewed and approved by Ethikkommission der Universität Ulm Helmholtzstraße 20 (Oberer Eselsberg) Protocol Nr: 66/20. The patients/participants provided their written informed consent to participate in this study.

## Author Contributions

The authors confirm contribution to the paper as follows: study conception and design: JK, CB, WL, ME. Data collection: JK, AJ, LB. Analysis and interpretation of results: JK, WL, LB. Draft manuscript preparation: JK, LB, WL, AJ. All authors reviewed the results and approved the final version of the manuscript.

## Funding

The Cryoprobes used in the study and the cryodevice (Erbekryo) were provided by Erbe Elektromedizin, Tübingen, Germany free of charge. The processing costs of the ethics application before the local ethics board were covered by the company Erbe Elektromedizin, Tübingen, Germany. The funder was not involved in the study design, collection, analysis, interpretation of data, the writing of this article or the decision to submit it for publication.

## Conflict of Interest

ME and WL are employed by Erbe Elektromedizin GmbH, Tuebingen Germany.

The remaining authors declare that the research was conducted in the absence of any commercial or financial relationships that could be construed as a potential conflict of interest.

## Publisher’s Note

All claims expressed in this article are solely those of the authors and do not necessarily represent those of their affiliated organizations, or those of the publisher, the editors and the reviewers. Any product that may be evaluated in this article, or claim that may be made by its manufacturer, is not guaranteed or endorsed by the publisher.
